# *Aedes albopictus* Populations and Larval Habitat Characteristics across the Landscape: Significant Differences Exist between Urban and Rural Land Use Types

**DOI:** 10.3390/insects12030196

**Published:** 2021-02-25

**Authors:** Katie M. Westby, Solny A. Adalsteinsson, Elizabeth G. Biro, Alexis J. Beckermann, Kim A. Medley

**Affiliations:** 1 Tyson Research Center, Washington University in Saint Louis, 6750 Tyson Valley Road, Eureka, MO 63025, USA; solny.adalsteinsson@wustl.edu (S.A.A.); ebiro@wustl.edu (E.G.B.); lexiebeckermann@gmail.com (A.J.B.); kim.medley@wustl.edu (K.A.M.); 2 Department of Biology, Southeast Missouri State University, 1 University Plaza, MS 6200, Cape Girardeau, MO 63701, USA

**Keywords:** *Aedes albopictus*, mosquito, urban ecology, phenology, microclimate, detritus, nitrogen, pH, tannins, hydroperiod, heterospecifics

## Abstract

**Simple Summary:**

More than 50% of the global population now resides in cities. In addition to large human populations, some cities support large populations of vectors, such as mosquitoes. Consequently, we have seen a resurgence in urban outbreaks of mosquito borne diseases, e.g., West Nile and dengue fever, in the last 60 years. The Asian Tiger mosquito, *Aedes albopictus*, is an ecologically flexible species that has colonized every continent, except Antarctica, in recent decades. In this paper, we tested the prediction that *A. albopictus* is more abundant in human-dominated, urban, and suburban areas compared to rural areas in a temperate US city. We collected data on mosquito abundance and other aspects of the environment important for mosquitoes and that also may vary by land use type. As predicted, we found higher densities of *A. albopictus* in urban and suburban areas compared to rural ones. We also found few other mosquito species, higher average temperatures, lower nitrogen levels in aquatic larval habitats, and faster water evaporation in some sample weeks. We conclude that this species is thriving in human-dominated areas in this metropolitan region, and its success may be partially due to the environmental characteristic of this habitat type.

**Abstract:**

One of the most profound recent global changes has been the proliferation of urban metropolitan areas. A consequence of urbanization is a reduction in abundance, or diversity, of wildlife. One exception, is the proliferation of vectors of disease; recent years have seen the emergence and resurgence of diseases vectored by species closely associated with humans. *Aedes albopictus*, a mosquito with a near global range and broad ecological niche, has been described as an urban, suburban, or rural vector, or a forest edge species depending on local conditions. We tested the hypothesis that abundance and phenological patterns of this species vary among different land use types in a temperate city because of the variation in the biotic and abiotic conditions characteristic of those habitat types. *A. albopictus* populations in urban and suburban areas were an order of magnitude larger than in rural areas and were detected several weeks earlier in the season. Additionally, we found fewer overall mosquito species, higher temperatures, lower nitrogen, higher pH, and faster water evaporation in larval habitats in urban vs. rural areas. By understanding the ecological differences that facilitate a species in one habitat and not another, we can potentially exploit those differences for targeted control.

## 1. Introduction

The mass migration of people from small towns and rural areas into urban areas has been one of the most profound changes to the global landscape [1]. The concentration of people in highly built environments has created a globally replicated, human-made habitat type with no historical analog. Urban areas can be characterized by high human densities, large expanses of impervious surfaces, and an abundance of artifacts and refuse associated with modern humans. Urban habitat has long been viewed as inferior to “undisturbed” and “natural” habitat for wild flora and fauna and is often associated with local extirpation of species or declines in species richness and biodiversity [2,3]. The abundant refuse, however, has created novel resources leading to some species adapting to, and thriving in, the human-dominated landscape [4,5]. Importantly, many of these species capitalizing on the refuse are considered pests or are vectors of human and domestic animal disease [6,7]. While urban areas typically have low vector diversity [8,9], they correlate with a proliferation of the most notorious vectors of disease.

Disease transmission in urban areas is in step with increases in human density. Diseases such as dengue and chikungunya have resurged, and newly emerged viruses like West Nile and Zika have caused global pandemics in the latter half of the 20th and into the 21st century [6,10,11]. Notably, the most important vectors of these viruses are all highly associated with humans (e.g., *Aedes aegypti*, *Aedes albopictus*, *Culex pipiens* complex) [12,13]. Mathematical models describing the transmission of these vector-borne viruses incorporate the density of vectors as important drivers of outbreaks [14,15]. It is, thus, important to explore the aspects of the human-dominated, urban landscape that allow these vectors to thrive.

At broad spatial scales, climate largely determines the geographic range of mosquito vectors [16]. At local scales, however, abundance patterns are influenced by a suite of abiotic factors: availability of larval habitats [17], shade for resting [18], and microclimate [19,20,21], as well as biotic factors: availability of blood hosts [22,23] and nectar sources [24,25], predator density [26], competitors [27], and microorganism resources in larval habitats [28]. These environmental factors are known to influence all of the components of vectorial capacity models and population growth estimates for mosquitoes [29,30]. Furthermore, there is evidence that these factors also vary with urbanization intensity, e.g., microclimates [21]. Other aspects of the landscape, such as vegetation type and cover, will impact vector abundance [31]. For example, ornamental vegetation in gardens [32,33], or overgrown vegetation from abandoned lots [34,35,36] provide shade, nectar, humidity, and fast-decomposing detritus inputs [37] into larval habitats that may not be as available in more sylvan habitats [38]. Additionally, environmental conditions in urban areas can influence litter decomposition rates, nutrient composition, and microbial growth in vegetation that have the potential to alter its quality as detritus in larval habitats [39,40,41]. The amount and type of litter that falls into aquatic larval habitats as detritus forms the base of the food web and strongly affects the number of mosquitoes a container can support [42]. Additionally, soluble nutrients in aquatic habitats (e.g., nitrogen) may be limiting factors for growth [43], and chemicals, such as tannins [44], or acidification [45] can negatively affect growth or cause larval mortality. Container hydroperiod, or the length of time a container holds water before drying out, has important implications for larval survival to adulthood, detritus quality, and predator abundance [46,47,48,49] and is likely to be influenced by microclimatic conditions. It is unknown, however, if the quality of artificial larval habitats varies along an urbanization gradient in terms of resource availability, water quality, or length of hydroperiod.

In this paper, we focus on *A. albopictus* in a temperate US city (Saint Louis, MO, USA) and its surrounding suburban and rural areas. *A. albopictus* is a globally invasive species capable of transmitting at least 26 known viruses and *Dirofilaria immitus*, the parasite responsible for dog heart worm [50]. Since its establishment in the USA in 1985, this species has expanded its range to cover most of the eastern half of the USA and more recently parts of Southern California [51,52]. It is an aggressive day biting mosquito and is a considerable nuisance in areas where it reaches high densities [53]. Throughout its global range, this species occupies different ecological niches and has been described as an urban vector [54], a more suburban or rural vector [55], or a forest edge species [56] depending on local conditions. We tested the hypothesis that *A. albopictus* larval population abundance and phenological patterns will differ among three categorical land use types (urban, suburban, and rural), because attributes of artificial larval habitats will differ among these land use types. Specifically, we measured the abundance of potential competing mosquito species and predators, three water chemistry variables as proxies of habitat quality, weekly water evaporation rates, and microclimates directly next to our sample locations.

## 2. Materials and Methods

### 2.1. Sample Design

**Figure 1:**
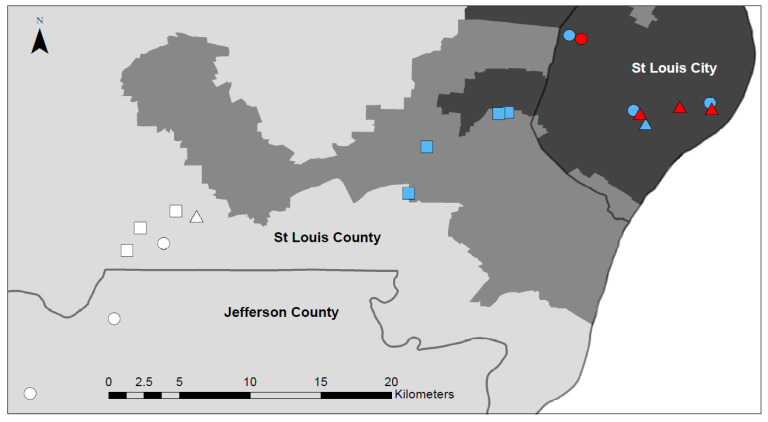
Mosquito sampling sites by habitat type. Rural: light grey, Suburban: Medium grey, Urban: dark grey. Squares: site was sampled in both years, Circle: site was sampled in 2017, Triangle: site was sampled in 2018. The color of the icons denote the percent impervious for each site at a 30 m^2^ scale (data obtained from the Multi-Resolution Land Characteristics Consortium). White: <5%, Blue: 5–55%, Red: >55%. County boundaries indicated with dark grey lines.

We sampled from four replicate oviposition cups placed at each of four urban sites, four suburban sites, and four–six rural sites (six in 2017 and four in 2018). In 2017, four of the rural sites were located at Tyson Research Center, a 2000-acre field station, and two at rural residences. The urban and suburban sites were private residences or businesses. In 2018, we had four sites in each of the three land use categories; three of the rural sites were at Tyson Research Center and one at an adjoining park. All urban sites were located within the city limits of Saint Louis with human population density ≥1500 individuals per km^2^. Suburban sites were located in townships in Saint Louis County along the Interstate 44 corridor with populations densities that ranged from 1000–1499 per km^2^. Rural sites had population densities ranging from ≤1000 per km^2^ (Figure 1). We used the Missouri House Districts map for population density (https://data-msdis.opendata.arcgis.com/datasets/mo-2011-house-districts?geometry=-91.715%2C38.383%2C-89.629%2C38.758&orderBy=BLACK&orderByAsc=false (accessed on 10 October 2020)). In both years, four cups were placed at each site for a total of 56 individual cups in 2017 and 48 in 2018. All sites were 1 km or more apart, which is estimated to be the maximum lifetime natural dispersal of *A. albopictus* [57]. Mosquito larvae were sampled weekly in 2017 (20 June–5 September) and 2018 (23 May–4 September) from 1 L black plastic oviposition cups. The cups were deployed in the field on 7–12 June in 2017 and 12–13 April in 2018 and subsequently monitored for oviposition activity; samples were collected after larvae were first detected in the cups. Each cup was filled to capacity weekly with rain water but detritus was allowed to accumulate naturally with no initial additions. All cups were attached to fences or vegetation, ranging from ground level to waist height in full to partial shade. To facilitate sampling, we used a two-cup system where an outer cup with holes drilled in the top was attached with a zip-tie, with a drain hole in the bottom, and a second cup with no holes was placed inside. This allowed us to remove the inner cup and pour the entire contents into a white dishpan, remove all larvae, and return the water and detritus to the cup easily. All larvae and pupae were removed with a pipette and returned alive to the lab where they identified two species weekly. First instar larvae and pupae were housed in an environmental chamber (25 °C 16:8 L:D) until they were second instars or larger or had ecolosed as adults, for identification.

### 2.2. Water Chemistry and Evaporation

After the final larval sample was collected in 2017, the cups were sealed with food-grade plastic wrap, and the entire contents were returned to the lab. We homogenized the water column and then took a sample to measure total nitrogen, total tannins (TN), and pH. TN and tannins were quantified using the Hach DR 2800 spectrophotometer (Loveland, CO, USA) following the manufacturer’s protocols for Method 10071 for TN and Method 8193 for tannins. pH was measured using Eutech Instruments ecoTestr pH1 (a division of Thermo Scientific Inc., Waltham, MA, USA).

We also measured total water volume from each cup weekly in 2018 to assess potential differences in evaporation between land use types. All cups were attached to trees, posts, or vegetation and were thus not all completely vertical leading to small differences in the maximum water volume. To account for these differences, we measured the maximum water volume for each individual cup and the water volume weekly before taking the larval sample and refilling the cup with rain water to its maximum volume. We were then able to calculate the weekly percentage water loss.

### 2.3. Temperature and Humidity

In 2018, we also recorded the microclimate at each site using a HOBO temperature and humidity logger placed directly next one of the cups (Onset HOBO MX2301, Bourne, MA, USA). Recordings were made every three hours from 12 April–3 September. It is well established that temperatures in urban areas are higher than those in more suburban and rural areas. Less is known about the extent of microclimactic differences collected at *A. albopictus* breeding sites (but see [58]). We summarized our temperature data as overall daily mean temperatures and as a histogram of the frequency, in days, that temperature differences were biologically relevant to this species (<1, 1–2, >2 °C) and daily mean relative humidity.

### 2.4. Statistical Analyses

#### 2.4.1. *A. albopictus* Abundance

We analyzed differences in *A. albopictus* abundance with a generalized linear mixed model (GLMM), using the negative binomial error distribution, with year, land use type (urban, suburban, rural), sample week, and all two-way interactions as categorical main effects with site as a random effect. *A. albopictus* abundance from the four individual cups at each site were summed for a single value per site, per sample week. We included sample week as a main effect, as we determined *a priori* that abundances would non-randomly change through time based on phenological patterns from previously published studies [59,60]. For this formal statistical analysis, we only included the 12 weeks that were sampled in both years (20 June–5 September). We also ran separate analyses for both years to obtain least squares means with the full 2018 data set and explore potential phenological differences in *A. albopictus* abundance between land use types. There were very few observations of any species other than *A. albopictus* in urban and suburban cups, so no formal statistical tests were performed on these species. Instead we present a table of the total numbers collected of each species by land use type (Table 1).

#### 2.4.2. Water Chemistry and Evaporation

For the 2017 water chemistry data, which were only quantified once after the final larval sample, each cup was treated as an independent observation (56 total observations). While there were four cups placed at each site, the cups were all placed in, or under, different vegetation types and considered independent replicates of the possible larval habitat conditions created in each land use type. Each of the three variables (TN, tannins, and pH) were analyzed separately using a GLMM with land use type as a fixed effect and site as a random effect. Error distributions were chosen for each variable based on the best fit of the data; TN was analyzed using the lognormal error distribution, tannins and pH with the normal distribution. Tukey’s correction for multiple comparisons was used where appropriate. We also ran a simple stepwise linear regression to determine if there was a relationship between the abundance of *A. albopictus* from each cup, summed over the entire sampling period, and either of the three water chemistry variables measured.

In 2018, water levels were recorded in each cup weekly. As with the water chemistry data, each cup was treated as an independent replicate. Percent water loss was analyzed with a GLMM with land use type, sample week, and the interaction as main effects and site as a random effect using the negative binomial error distribution.

## 3. Results

### 3.1. A. albopictus Abundance

**Figure 2:**
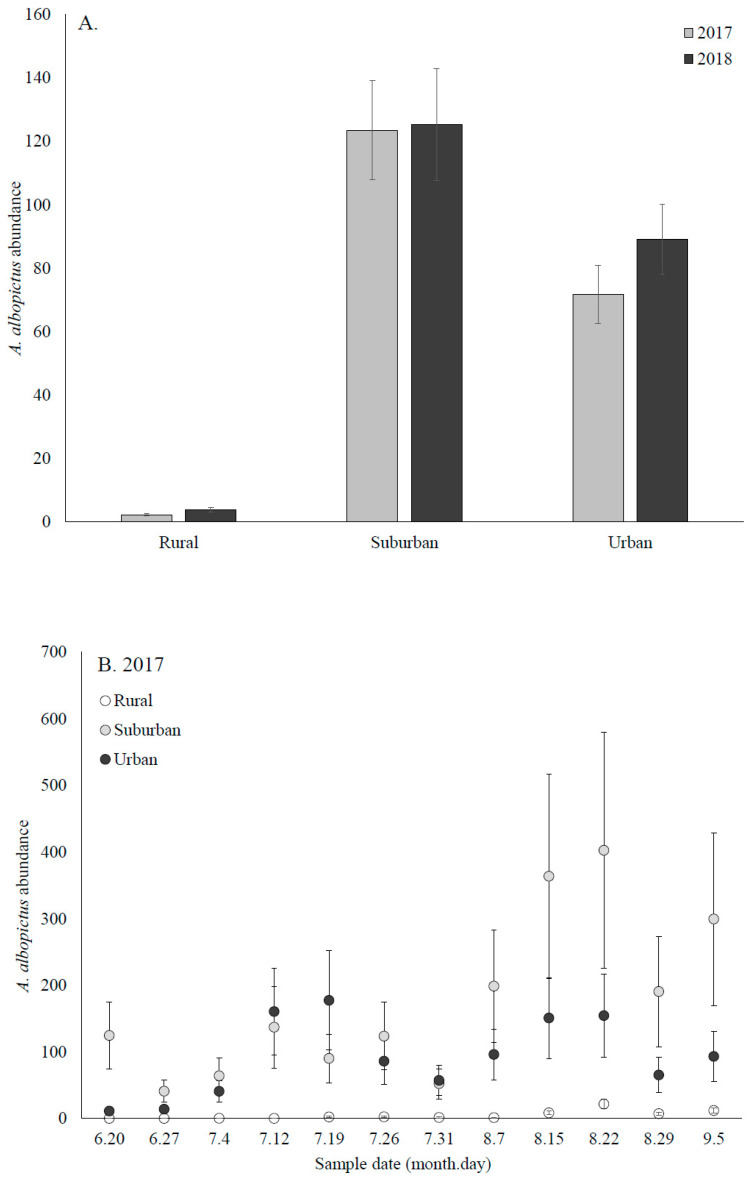

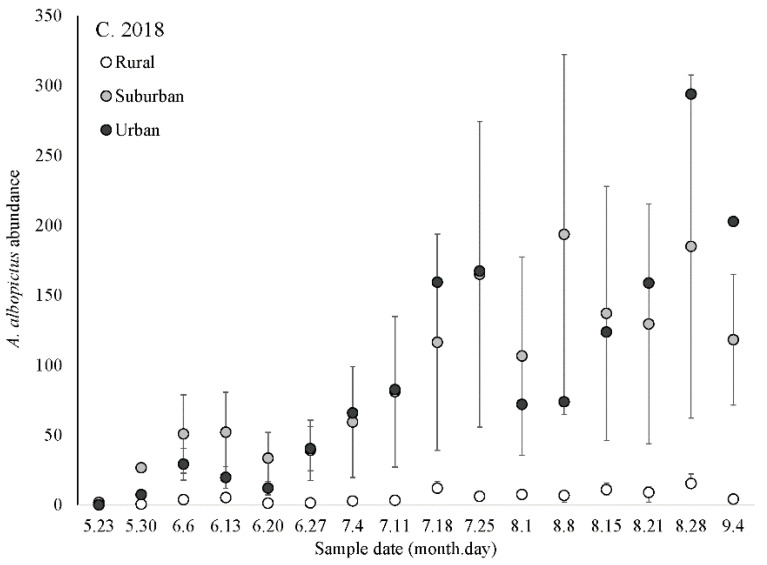
Abundance of *A. albopictus* larvae collected from oviposition cups. (**A**) The interaction between land use type and sample year. (**B**) The interaction between sample week and land use type in 2017 and (**C**) 2018.

*A. albopictus* was significantly more abundant in urban and suburban sites, which did not differ from each other, than rural sites (Figure 2A). Our analyses showed significant effects of year (F_1,246_ = 5.11, *p* = 0.0247), land use type (F_2,246_ = 241.65, *p* < 0.0001), sample week (F_11,246_ = 21.63, *p* < 0.0001), the interactions between year and sample week (F_11,246_ = 2.55, *p* = 0.0045), and land use type and sample week (F_22,246_ = 3.11, *p* < 0.0001), but not year and land use type (F_2,246_ = 1.71, *p* = 0.1830). While there was variation between years, land use types, and sample weeks (Figure 2B,C), the overall pattern was consistent across the two study years. Additionally, there was a comparative delay in detecting *A. albopictus* at rural sites in both years (Figure 2B,C).

There were very few instances of any other mosquito species being collected from suburban and urban cups. In 2018, several hundred *Culex* individuals were collected, but these were isolated events in which two individual cups had large inputs of floral detritus fouling the water and attracting *Culex* [61]. Even in the rural cups, few of the predatory species *Toxorhynchites rutilus* were collected (Table 1).

### 3.2. Water Chemistry and Evaporation

**Figure 3:**
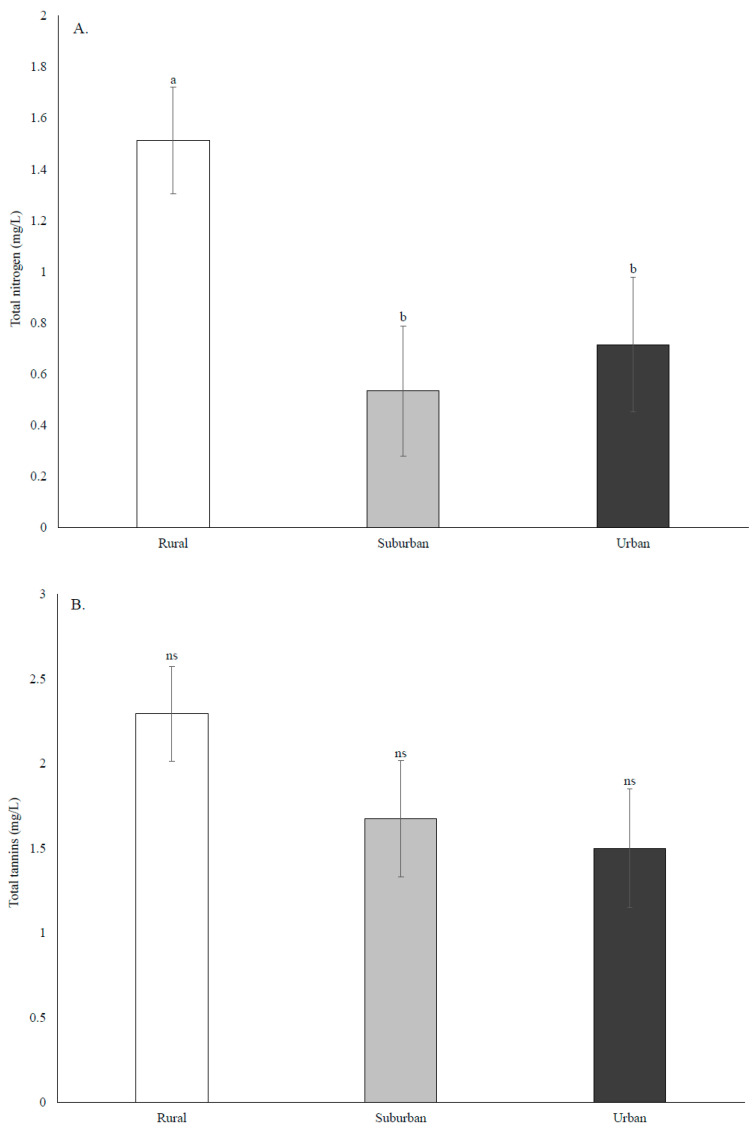

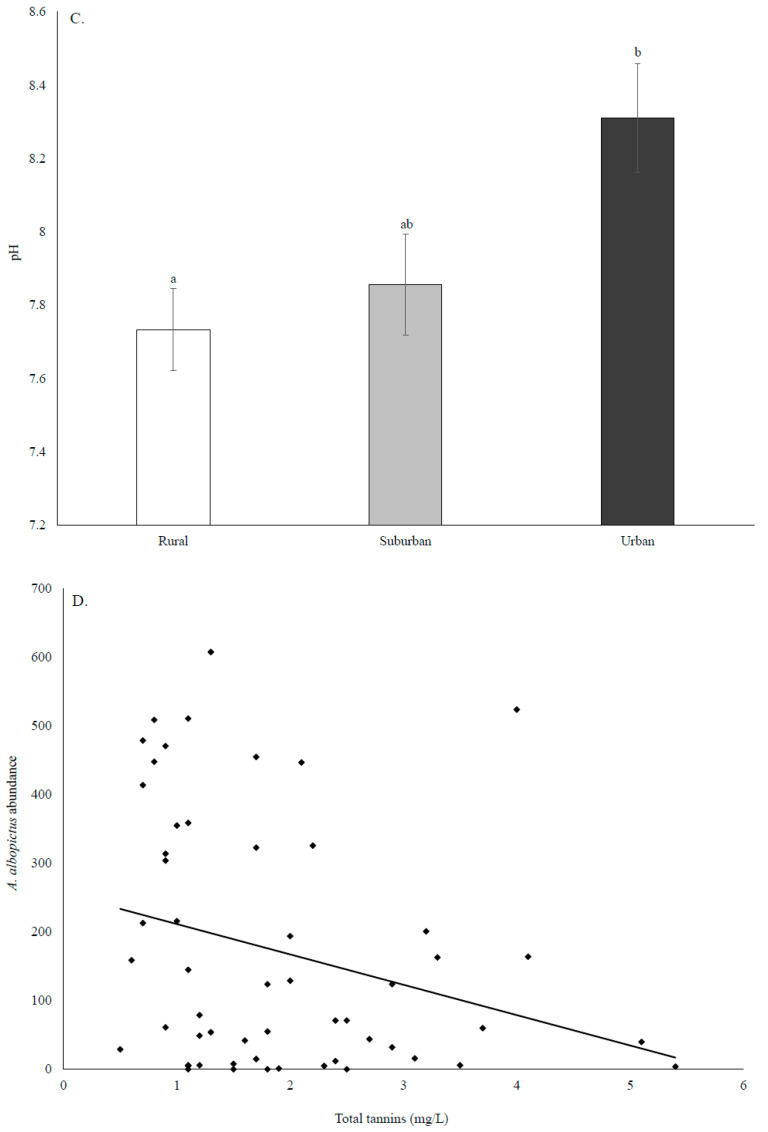
Water chemistry measured at a single time point at the end of the sampling period in September 2017. (**A**) Total nitrogen, (**B**) total tannins, (**C**) pH, and (**D**) the negative relationship between the total *A. albopictus* collected from a cup and the total tannins measured from that cup. Different lower case letters above bars indicate groups that are significantly different from each other.

There was a significant effect of land use type on TN (F_2,41_ = 5.30, *p* = 0.0090). Higher levels of TN were detected in rural cups compared to urban and suburban cups, which did not differ (Figure 3A). The effect of land use on tannins was not significant (F_2,41_ = 1.87, *p* = 0.1670) (Figure 3B); however, there was a significant effect on pH (F_2,39_ = 5.01, *p* = 0.0116). The pH was significantly higher in urban cups compared to rural cups, but the other comparisons were not significant (Figure 3C). The stepwise regression model included the effects of TN (F = 2.59, *p* = 0.1140) and tannins (F = 5.19, *p* = 0.0269) with an adjusted R^2^ of 0.1026. There was a slight, but significant negative relationship between *A. albopictus* abundance and tannin concentration (Figure 3D).

**Figure 4:**
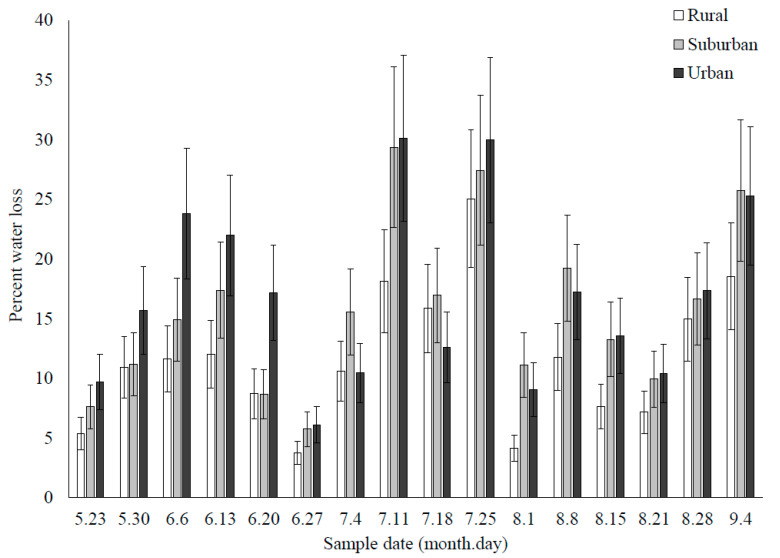
Ovicup hydroperiod, measured as weekly percent water loss in 2018.

The main effect of land use type on percent water loss was not significant (F_2,691_ = 1.22, *p* = 0.2970); however, the effect of sample week (F_15,691_ = 30.00, *p* < 0.0001) and their interaction were significant (F_30,691_ = 5.01, *p* = 0.0078) (Figure 3). There was a lot of within land use type and weekly variation in the percent of water lost from the cups, but in general, water loss was lower in rural sites compared to urban sites (Figure 4).

### 3.3. Temperature and Humidity

**Figure 5:**
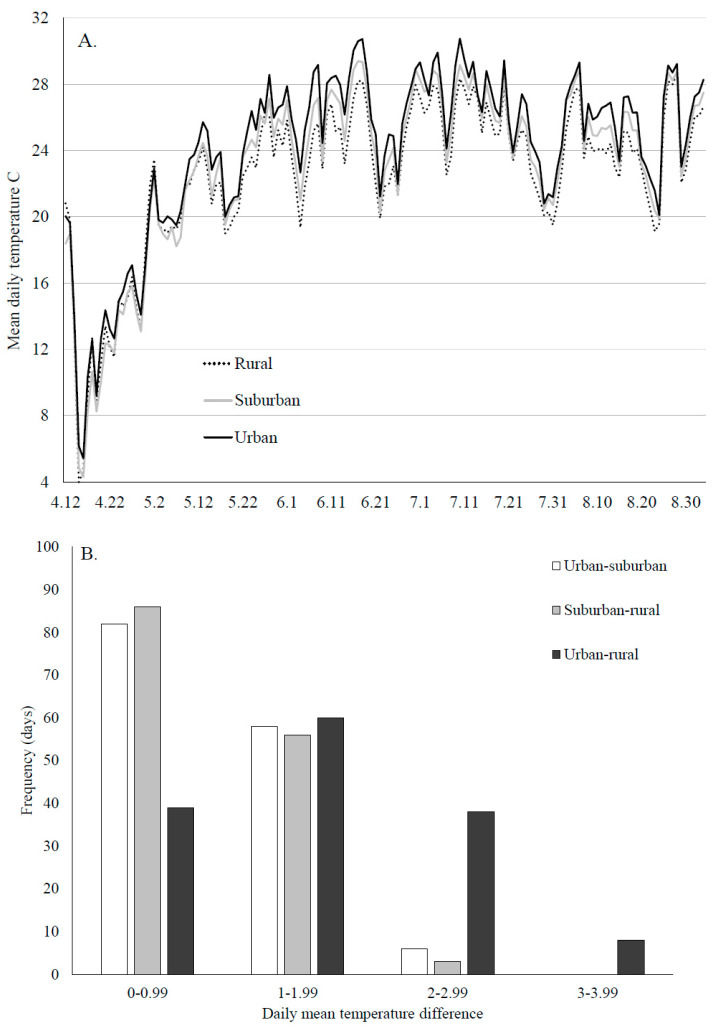

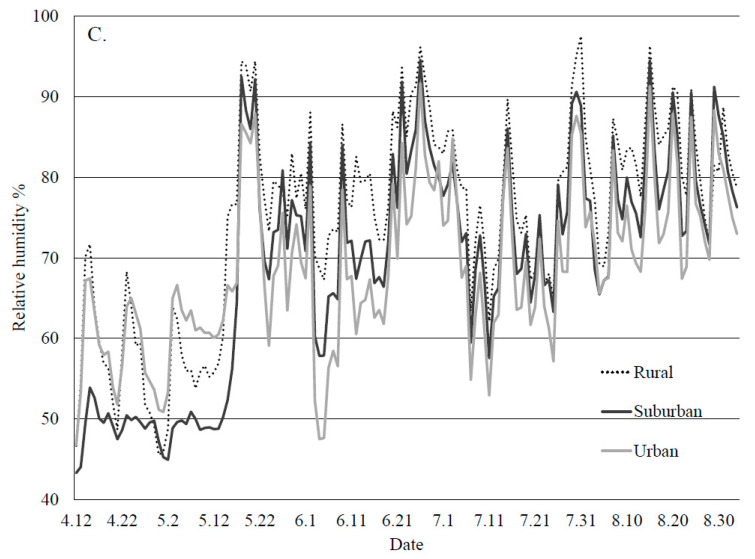
Temperature and humidity were measured every three hours at each site, directly next to one of the ovicups, in 2018. (**A**) Daily mean temperatures by land use type, (**B**) a histogram of the frequency that differences in daily mean temperatures between each land use type reached levels that may be sufficient to alter population growth rates, (**C**) daily mean relative humidity.

As expected, daily mean temperatures were generally higher in urban areas compared to rural ones with suburban temperatures being intermediate (Figure 5A). We were specifically interested in the number of days throughout the mosquito breeding season that temperatures, measured at our larval sampling sites, differed in a magnitude large enough to alter development times and population growth estimates. We estimated that threshold to be 2 °C based on a laboratory study [62]. On the majority of the 145 days we recorded, daily mean temperature differences between land use types were less than 2 °C, indicating that for the majority of the spring and summer, temperature differences, when measured at the microclimatic scale, may not be large enough to affect growth rates. The largest temperature differences occurred between urban and rural sites (Figure 5B). Daily mean humidity was also highly variable throughout the season. The greatest differences between the land use types occurred in the spring before any mosquitoes were collected. In general, humidity was highest in rural areas, and lowest in urban ones (Figure 5C).

## 4. Discussion

The notion that the density of mosquitoes is important for predicting disease risk is at least as old as the works of Ronald Ross at the turn of the 20th century [63]. It has thus been a century long pursuit to understand the factors that determine mosquito population densities at a relevant scale, particularly for important vector species. The world’s most important arbovirus vectors, and the viruses they transmit, are all closely associated with human habitation [6]. *A. albopictus* has achieved a nearly global distribution in recent decades, is an aggressive biter often reaching high densities, and vectors important viruses such as dengue and chikungunya [50]. In this paper, we present data that show that *A. albopictus* population abundance and phenology varies among three land use types. We also show that there are significant differences in artificial larval habitats; specifically, abundance of heterospecifics, water chemistry, microclimate, and hydroperiod.

We found that *A. albopictus* larval abundance was as much as an order of magnitude higher in suburban and urban cups compared to rural ones. There was also a significant delay in detecting *A. albopictus* larvae in rural cups in both years we sampled. Additionally, the general increase in abundance we observed throughout the growing season in suburban and urban cups was not achieved in rural ones, and densities always remained comparably low. This fits with the pattern observed at Tyson Research Center (one of our rural sample sites); despite a decade plus of establishment in this forest, *A. albopictus* is not a dominant species in artificial containers [64]. Differences in abundance by urban–rural land use classifications have been documented for this species in other geographic areas. Interestingly, there is not a consistent overall pattern throughout its global range. For example, populations are larger in urban areas in Guangdong Province, China, while suburban and rural populations are smaller and more similar in size [54]. In Brazil, *A. albopictus* is most abundant in peri-urban and sylvan areas or urban parks and is much less associated with urban dwellings [65]. Two major issues arise, however, when comparing patterns among regions and studies. First, researchers use different criteria to qualify land use types that are not easily comparable [66]. Second, there are many other confounding environmental variables that can predict abundance that are not accounted for, nor would it be possible to account for, in each study. One such example is neighborhood socio-economic status, which is a significant predictor of *A. albopictus* abundance in Baltimore, Maryland, and Washington, DC [67], but not in southeastern New York State only a few hundred kilometers away [68]. Additionally, the strength of interspecific interactions among species will vary depending on the species present [69]. *A. albopictus* abundance may be more depressed in urban areas when *A. aegypti* is present, such as in Brazil and Florida, USA [65,70]. This global variation in relative densities by habitat type makes it even more crucial to understand density patterns to inform local vector control methods.

In the Saint Louis, Mo region, we found that *A. albopictus* is the dominant species occupying ovicups in urban and suburban areas. In fact, outside of a few individuals of other *Aedes* spp. and a few instances where the water became foul due to a large input of ornamental flowers attracting *Culex* [61], it was the sole species collected. This is in contrast to rural habitat, where other native and non-native species were present, consistent with the hypothesis that human-dominated landscapes have lower vector diversity. Based on the data from our 1 L ovicups, it appears that these species are rare in, or have been extirpated from, metropolitan Saint Louis. Container breeding species, however, have preferences for different sized vessels [46], so it is possible that we missed other species by using only one size of cup for our samples. In fact, we have collected many *Culex* spp. from 8 L buckets and tires in the area and have seen the predatory *T. rutilus* in tree-holes in urban parks (Westby, *unpublished data*). Importantly, we did collect seven species using the 1 L cups at our rural sites, so we conclude that in many artificial containers in the human dominated landscape, *A. albopictus* faces little to no interspecific competition or predation. Saint Louis has had an established *A. albopictus* population likely since 1986, one of the earliest cities in the USA [71]. From the limited information available in the literature about the species that occupied containers in temperate metropolitan areas before the infestation, it appears that *Aedes altropalpis*, *Aedes triseriatus*, *Culex pipiens*, and *Culex restuans* were the dominant species [72,73,74]. It is possible that *A. albopictus* is responsible for the local extinction of other *Aedes* spp. in suburban and urban areas, something that it has not been able to accomplish in rural and sylvan habitat [64]. This supports our hypothesis that artificial containers have different biotic communities across the landscape. Future work is needed to further elucidate these findings and should include surveys and experiments using a wide range of container types and sizes (e.g., natural vs. artificial, tires vs. buckets) to fully characterize the container mosquito community across the landscape.

We also hypothesized that the abiotic conditions within artificial container habitats would differ among land use types. Microclimates at breeding and resting sites have been shown to be important in estimating population growth rates and vectorial capacity for *A. albopictus* in Georgia, USA [21,58]. In our study area, as expected, we found that temperatures were generally higher at urban sites compared to rural ones, with suburban sites intermediate. Most of the differences in daily mean temperatures between land use types were less than 2 °C, which may or may not be sufficient to impact growth rates. Laboratory studies examining the impact of temperature on growth rates in this species test at temperature intervals of 2–5 °C [62,75,76]. The mean temperature difference between rural and urban sites was greater than 2 °C for more than 40 days during our sampling period, which, based on laboratory studies [62], may be sufficient to increase the number of generations in a growing season for this multivoltine species. We also detected generally higher relative humidity in rural compared to urban habitat. Much less is known about how humidity affects *A. albopictus* (but see [77]) outside of its effects on rates of egg desiccation [78], which can be severe enough to limit population growth and impact the outcome of interspecific competition [79]. It does not appear that the humidity is low enough in our urban and suburban microclimates to have a deleterious effect, as *A. albopictus* populations are thriving.

Evaporation from cups was highly variable among land use types, likely driven by highly variable daily mean temperatures and humidity, or rainfall (which we did not measure). There was a significant interaction between sample week and land use type, highlighting the temporally and spatially dynamic nature of larval habitats across the landscape. In some weeks, the water loss was greater in urban vs. rural areas, and it was equivocal in other weeks. Even in weeks with overlapping errors, urban cups lost as much as 10% more water than rural cups. This could potentially lead to differences in larval growth rates [48], total habitat desiccation, especially for smaller sized containers [46,80], and differences in detritus and solute concentrations [81]. Future research is needed to explore the importance of differences in water evaporation rates across the landscape.

The importance of detritus type and amount, and soluble nutrients, for container communities is well established [82,83,84]. Detritus, which forms the base of the food web in these aquatic communities, largely determines the number of mosquito larvae a container can support to adult emergence, the outcome of interspecific competition, and even the distribution of species on a local scale [28,37,60]. Much less is known, however, about how land use type affects the quality of container habitats through effects on detritus composition and quality. Two notable exceptions found that land use type had significant effects on detritus amount, water chemistry, and mosquito community composition (urban vs. pasture [85], forested vs. unforested [86]), though these significant differences were not found for the entire breeding season [86]. In our study, we found that total nitrogen was higher in rural cups compared to suburban and urban cups; the opposite was true for pH. Similarly to Kling et al. 2007 and Leisnham et al. 2007, we found limited evidence that water chemistry was a predictor of mosquito abundance. Interestingly, the significant correlations that were observed contrasted from previous work. In one study there was a significant correlation with pH [85], phosphorus in another [86], and tannins in the present study. We observed a negative correlation between *A. albopictus* abundance and tannin concentration. This result is unsurprising, as tannins are generally considered detrimental to aquatic insects, precipitating protein [87], damaging gut tissues [88], prolonging larval development, and increasing mortality rates [44]. We were, however, only able to measure water chemistry once at the end of the study, so any fine-scale or temporal variation in these metrics was not captured.

It appears that, in general, water chemistry may be a poor predictor of larval mosquito abundance in the field, and in turn, of larval habitat quality. Previous work in laboratory studies has shown that nitrogen, tannins, and pH can significantly affect the performance of *Aedes* mosquitoes [43,44,45,83,89]. For example, the addition of soluble nitrogen had a significant positive effect on larval survival and adult mass [43], but was not a significant predictor of *A. albopictus* abundance in tire samples [90], suggesting that while nitrogen is important in the larval habitat, it may not vary on a biologically relevant scale in field habitats or is not limiting enough to be correlated with abundance. The abundance of mosquito larvae in individual samples is not a direct measure of habitat quality and may not accurately reflect the number of adult mosquitoes a container can produce. Nutrient ratios (C, N, and P), however, were significant predictors of larval abundance in field containers in Florida, USA, and mosquito survival in follow-up laboratory bioasssays [37], suggesting that nutrient ratios may have more explanatory power than absolute numbers. Recognizing the weaknesses in extrapolating habitat quality from a single point measurement of water chemistry, future work should include additional bioassays of habitats created in different land use types to determine if there are meaningful differences in terms of population growth potential.

## 5. Conclusions

In 2018, the CDC released a report indicating that reported cases of vector-borne diseases tripled between 2004 and 2016 (https://www.cdc.gov/vitalsigns/vector-borne/index.html (accessed on 4 January 2021).). With the climate changing, and increasing uncertainty as to how that will affect the incidence, and new emergence, of vector-borne diseases, it is increasingly urgent that we explore the different factors that allow vectors to thrive. In this paper, we have demonstrated that an important vector species in a temperate metropolitan area shows distinct abundance and phenological patterns in rural areas compared to urban and suburban ones. This species thrives in the human dominated landscape in this region, potentially putting thousands of residents at risk. Additionally, we have shown that there are constitutive differences between artificial container characteristics among land use types, notably the presence of heterospecifics, water evaporation rates, microclimate, and some water chemistry variables. We conclude that it is important to more rigorously explore the suite of variables that keep *A. albopictus* populations low in rural areas in this region in hopes of exploiting that knowledge for targeted control efforts.

## Figures and Tables

**Table 1:** The number of each species collected over the entire sampling period by land use type and year.

Year	Type	*A. albopictus*	*A. triseriatus*	*A. japonicus*	*A. hendersoni*	*C. restuans*	*C. pipiens*	*An. Barberi*	*T. rutilus*
2017	Rural	716	1019	36	79	0	0	14	6
	Suburban	5235	25	0	0	0	0	0	5
	Urban	3845	2	0	0	0	0	0	1
2018	Rural	519	2199	246	254	0	1	0	6
	Suburban	4873	2	9	48	616	268	0	2
	Urban	4867	7	0	13	159	651	0	0

## Data Availability

The data will be made publicly available in the Dryad Data Repository after publication of the manuscript.
